# Supplementary material for: Dynamics as a cause for the nanoscale organization of the genome
Source: Nucleus. 2020 May 23;11(1):83–98. doi: 10.1080/19491034.2020.1763093 (PMC7529413; doi:10.1080/19491034.2020.1763093)
Supplement: Supplemental Material [file KNCL_A_1763093_SM8360.zip › Supplementary information/Barth_et_al_supplementary_file.docx]

Supplementary Material

**Dynamics as a cause for the nanoscale organization of the genome**

Roman Barth ^1^, Genevieve Fourel ^2, 3^ and Haitham A. Shaban ^4, 5,^ *

1: Department of Bionanoscience, Delft University of Technology, 2628 CJ Delft, The Netherlands.

2: Laboratory of Biology and Modelling of the Cell, University of Lyon, ENS de Lyon, University of Claude Bernard, CNRS UMR 5239, Inserm U1210, Lyon, France.

3: Centre Blaise Pascal, ENS de Lyon, Lyon, France.

4: Spectroscopy Department, Physics Division, National Research Centre, Dokki, 12622 Cairo, Egypt.

5: Center for Advanced Imaging, Northwest Building, Harvard University, Cambridge, MA, 02138, USA.

*: To whom correspondence should be addressed: Haitham A. Shaban. Email: [h_shaban@g.harvard.edu](mailto:h_shaban@g.harvard.edu)

**Supplementary Note 1: Estimation of time and length scales to match theory and experiment**

**Time scale.** The time in Figure 5B is scaled by the Zimm time $\tau_{Z} \sim\eta_{s}R_{F}^{3}/(\sqrt{3\pi}k_{b}T)$, where $\eta_{s}$ is the solvent viscosity, $k_{b}T$ is the thermal energy and$R_{F} \sim\sqrt{N}b$ is the Flory radius of a Gaussian chain with $N$ the number of monomers and $b$ the bond length ^1^. We take twice the persistence length $l_{p}$ of DNA as an estimate for the bond length, where the persistence length is usually estimated between 50 and 200 nm ^2^, roughly in the order of $l_{p} \sim{10}^{2} nm$. Instead of taking into account the whole genome in our discussion, only sub-compartments thereof are considered which may correspond to the well-described topologically associated domains (TADs), or more generally to chromatin domains as defined for instance as chromosome subunits for genome replication, or yet to loops formed by either bridging or loop extruding factors ^3–6^. Such loops and domains can be hundreds of kilobases long (up to 1 or 2 Mb ^7^), and we will consider here a length of order $\sim{10}^{2} kb$.

The elementary structural subunit in chromatin is a nucleosome, consisting of DNA wrapped around a core nucleosome made of a histone octamer; a 145nt segment is wrapped around one core nucleosome, and a DNA linker up to 80nt separates two adjacent nucleosomes such that on average one nucleosome corresponds to a 190nt segment in the human genome, with significant possible deviation especially in gene regulatory regions. Therefore, we consider one stretched out nucleosome to be roughly a few tens of nanometers long. Considering the loop dimensions of ${10}^{2} kb$, there are in the order of ${10}^{3}$ nucleosomes involved, which would measure $\sim{10}^{4} nm$ when stretched out linearly, corresponding to about $\sim{10}^{2} l_{p}$. We can thus estimate the Flory radius as $R_{F} \sim{10}^{1} l_{p}$.

With the thermal energy of about $k_{b}T \sim4 pN\cdot nm$ and estimates of the viscosity inside the cell nucleus of the order of $\eta_{s} \sim{10}^{3} Pa\cdot s$ ^8^, we arrive at an estimate of the Zimm time of the order $\tau_{Z} \sim{10}^{5} s$. We can thus approximately convert the simulated time in units of the Zimm time in Figure 5B to absolute time in seconds and compare it with the experimentally accessible time scales.

**Length scale.** Following the considerations above, we recapitulate the order of magnitude for the persistence length $l_{p} \sim{10}^{2} nm$ and the contour length of chromatin loops *in vivo* $L \sim{10}^{4} nm$. The ratio $L/(2l_{p})$, which is commonly used in the theoretical treatment of active polymers ^9–11^, can thus be estimated to be in the range of $L/(2l_{p}) \sim{10}^{2}$. The two lines shown in Figure 5C correspond to the cases where indeed $L/(2l_{p}) \sim{10}^{2}$(light blue) and for a chromatin segment ten times shorter, for which $L/(2l_{p}) \sim{10}^{1}$ (dark blue).

**Magnitude of dynamics.** Active polymers are characterized by the dimensionless Péclet number $Pe=UL/D$, which determines, loosely speaking, the ratio between the advection and diffusion rate in a system ^12^. Here, $U$ is the linear flow velocity, $L$ is the length scale over which advection occurs and $D$ is the (passive) diffusion constant. For $Pe<1$, the system is diffusion-dominated, while the system is advection-dominated for $Pe>1$. To give an order of magnitude of the Péclet number of chromatin flow, one should be able to disentangle purely diffusive and activity-induced flows in living systems. To give a lower bound on the expected Péclet number, we consider the diffusion of chromatin under the influence of ATP-driven processes. Chromatin diffusion coefficients are found to be in the order of $D \sim{10}^{-3} \mu m^{2}/s^{\alpha}$^13^, while purely passive diffusion due to thermal fluctuations is expected to be lower ^14^. We next consider the advection rate induced by active processes. Let’s consider for instance transcription elongation by RNA polymerase II through a highly transcribed 100 kb long gene, which proceeds at maximum $U \sim2 kb/min$ *in vivo* ^15^ or equivalently $U \sim5 nm/s$ along a polymer with contour length $\sim10 \mu m$, which would form a coil of diameter $L \sim1 \mu m$. Note that transcribed chromatin loops are partially unfolded compared to the basic folding of chromatin and can expand over large distances, hence a putative length between 1 and 10 microns for a 100kb segment. This would result in a Péclet number in the order of $Pe \sim0.1- 1$. Another example is the loop extrusion activity by SMC complexes, measured *in vitro* to proceed at a speed of $U \sim0.5 kb/s \sim{10}^{2} nm/s$ ^4,6^ on naked DNA . Assuming that the speed of cohesin is in the same order *in vivo* when dealing with chromatin*,* such extrusion process acting on a segment with diameter $L \sim1 \mu m$ would yield a Peclet number $Pe \sim10-100$, following the same considerations as above. These two examples based on processive enzymes illustrate that the Péclet number is likely in the order $Pe \sim{10}^{-1}-{10}^{2}$ . Note however that *in vivo*, such processive motion of enzymes occur on top of a variety of active effectors which act in a pervasive manner and relentlessly animate the whole of chromatin in a nucleus - and not only selected regions as is the case of active genes when considering transcription by RNA polymerases.

The $Pe \sim{10}^{-1}-{10}^{2}$ range estimate can be therefore reasonably considered as applying to the whole of chromatin *in vivo*.

This range is considered as “biologically relevant regime” as indicated by a shaded area in Figure 5C.

**Supplementary Figures**

Supplementary Figure 1: Temporal regression weights and cross-correlation. A) For each unidirectional Granger-causal relationship, the average regression weight is shown over the first 10-time lags considered (only for those pixels for which the Granger-causal relationship was identified; error bars denote the standard error) (see Materials and Methods). Each predictor, conditional and target variable at time $\boldsymbol{t-}\boldsymbol{\Delta}\boldsymbol{t}$ can contribute to the value of the target variable at time $\boldsymbol{t}$, thus giving rise to 30 (3 variables and 10-time lags) regression weights per pixel. B) Normalized average cross-correlation between the predictor and target for the first 10-time lags as well as the conditional and target variable, respectively (correlation between target and target variable was omitted since the analysis focuses on the cross-correlation between variables).

Supplementary Figure 2: Additional parameter comparisons for the two cases that the NND causes the blob area (left) and vice versa (right). Related to and in the same style of Figure 4.

Supplementary Figure 3: The blob NND is more robust against merging in regions with a density of blobs than the blob area. Blobs on simulated images (as in ^16^) were segmented on the original simulated frame and a blurred version of it. The blobs in the blurred and unblurred frames were characterized by A) their area and B) their NND. The empirical cumulative distribution function (CDF) was computed C) for the area and D) the NND and the maximum difference between the CDFs of the blobs identified in the blurred and unblurred images is indicated. A lower $\boldsymbol{\Delta}$indicates that the distributions in A-B) are more similar. E) Additional useful quantities were computed to examine how blob area and NND react to a blob merging. Shows are the scaled mean differences of mean area and NND in the blurred and unblurred case, respectively. The scaling is over the overall mean area/NND on the left and over the overall standard deviation over the area/NND distribution on the right. The difference in NND between blurred and unblurred versions of the frames are consistently smaller than for the area, indicating that the NND is more robust against resolution-mediated merging of blobs than the area.

**Supplementary References**

1. Socol M, Wang R, Jost D, Carrivain P, Vaillant C, Le Cam E, Dahirel V, Normand C, Bystricky K, Victor J-M, et al. Rouse model with transient intramolecular contacts on a timescale of seconds recapitulates folding and fluctuation of yeast chromosomes. Nucleic Acids Res 2019;

2. Bouchiat C, Wang MD, Allemand JF, Strick T, Block SM, Croquette V. Estimating the persistence length of a worm-like chain molecule from force-extension measurements. Biophys J 1999;

3. Brackley CA, Taylor S, Papantonis A, Cook PR, Marenduzzo D. Nonspecific bridging-induced attraction drives clustering of DNA-binding proteins and genome organization. Proc Natl Acad Sci [Internet] 2013; 110:E3605–11. Available from: http://www.pnas.org/cgi/doi/10.1073/pnas.1302950110

4. Davidson IF, Bauer B, Goetz D, Tang W, Wutz G, Peters J-M. DNA loop extrusion by human cohesin. Science 2019;

5. Fudenberg G, Imakaev M, Lu C, Goloborodko A, Abdennur N, Mirny LA. Formation of Chromosomal Domains by Loop Extrusion. Cell Rep 2016;

6. Ganji M, Shaltiel IA, Bisht S, Kim E, Kalichava A, Haering CH, Dekker C. Real-time imaging of DNA loop extrusion by condensin. Science (80- ) 2018; 360:102–5.

7. Gibcus JH, Dekker J. The Hierarchy of the 3D Genome. Mol. Cell2013; 49:773–82.

8. Caragine CM, Haley SC, Zidovska A. Surface Fluctuations and Coalescence of Nucleolar Droplets in the Human Cell Nucleus. Phys Rev Lett 2018;

9. Eisenstecken T, Gompper G, Winkler RG. Internal dynamics of semiflexible polymers with active noise. J Chem Phys 2017;

10. Eisenstecken T, Gompper G, Winkler RG. Conformational properties of active semiflexible polymers. Polymers (Basel) 2016;

11. Martín-Gómez A, Eisenstecken T, Gompper G, Winkler RG. Active Brownian filaments with hydrodynamic interactions: Conformations and dynamics. Soft Matter 2019;

12. Incropera FP, De Witt DP. Fundamentals of heat transfer. 1981;

13. Shaban HA, Barth R, Bystricky K. Nanoscale mapping of DNA dynamics in live human cells. Bioarxiv 2018;

14. Zidovska A, Weitz D a, Mitchison TJ. Micron-scale coherence in interphase chromatin dynamics. Proc Natl Acad Sci U S A 2013; 110:15555–60.

15. Jonkers I, Kwak H, Lis JT. Genome-wide dynamics of Pol II elongation and its interplay with promoter proximal pausing, chromatin, and exons. Elife 2014;

16. Barth R, Bystricky K, Shaban HA. Coupling chromatin structure and dynamics by live super-resolution imaging. bioRxiv 2019; :777482.
