# Supplementary figures and images for: Dynamics as a cause for the nanoscale organization of the genome
Source: Nucleus. 2020 May 23;11(1):83–98. doi: 10.1080/19491034.2020.1763093 (PMC7529413; doi:10.1080/19491034.2020.1763093)

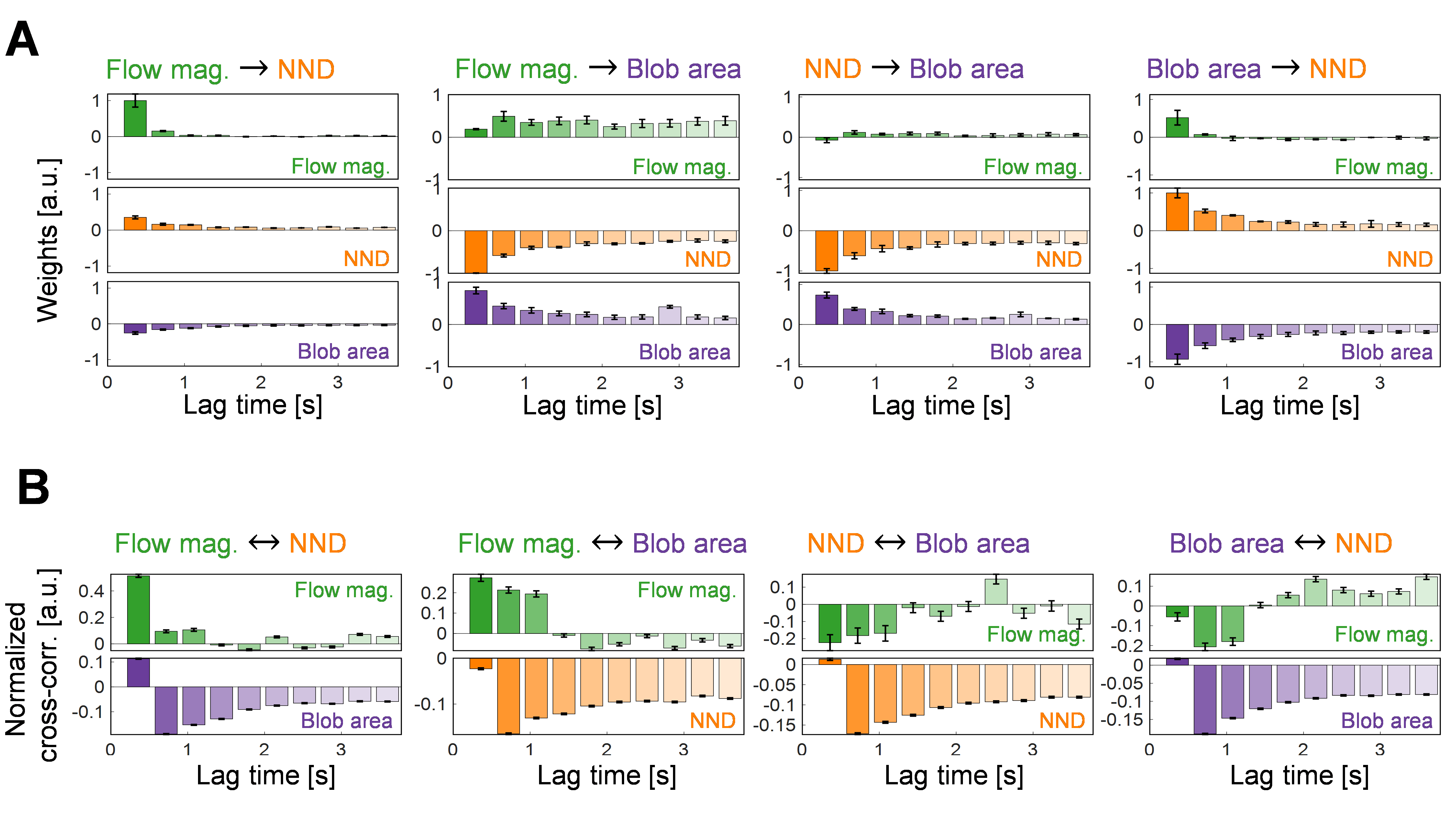

Supplement: Supplemental Material [file KNCL_A_1763093_SM8360.zip › Supplementary information/Figure_S1.png]

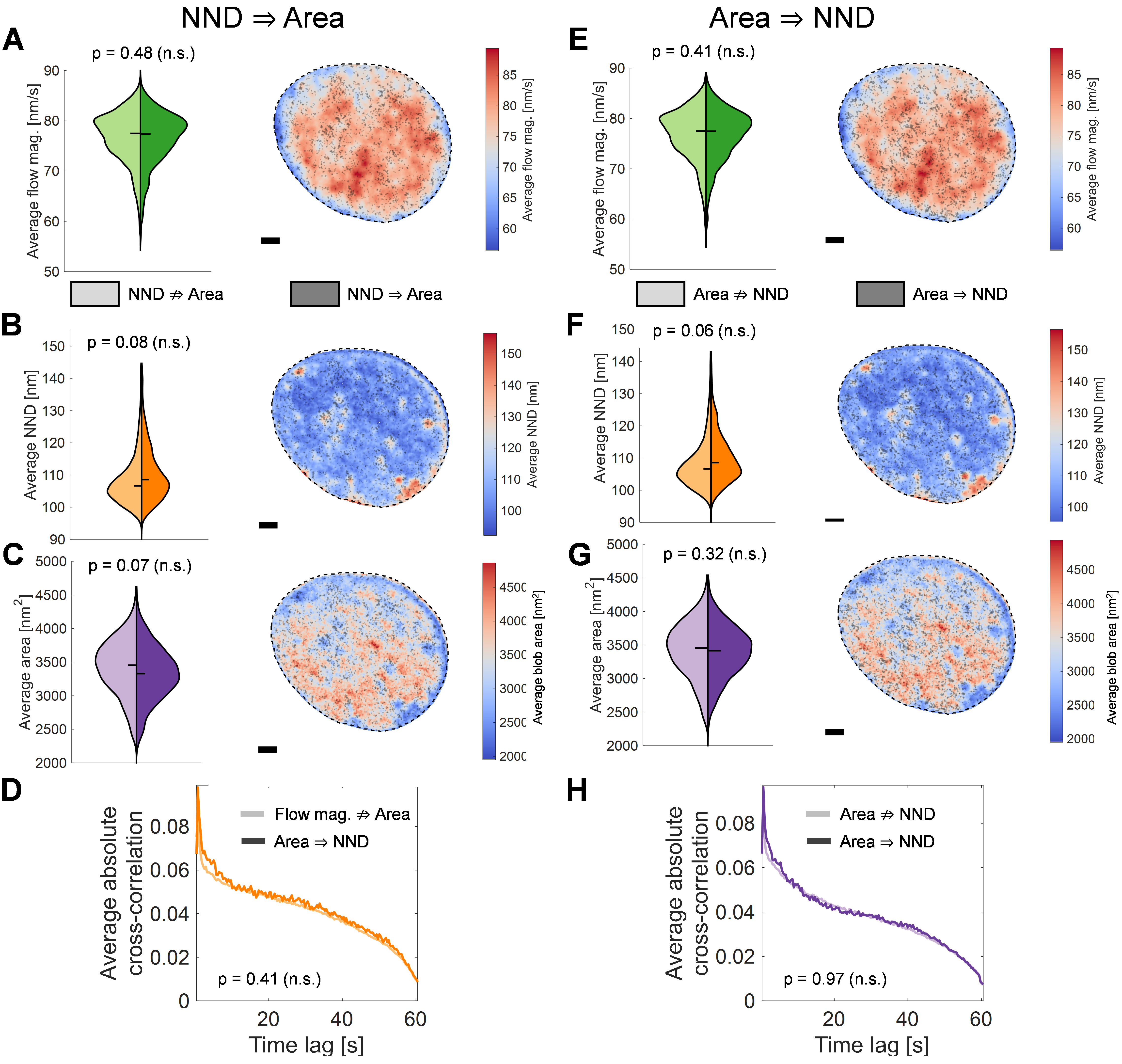

Supplement: Supplemental Material [file KNCL_A_1763093_SM8360.zip › Supplementary information/Figure_S2.png]

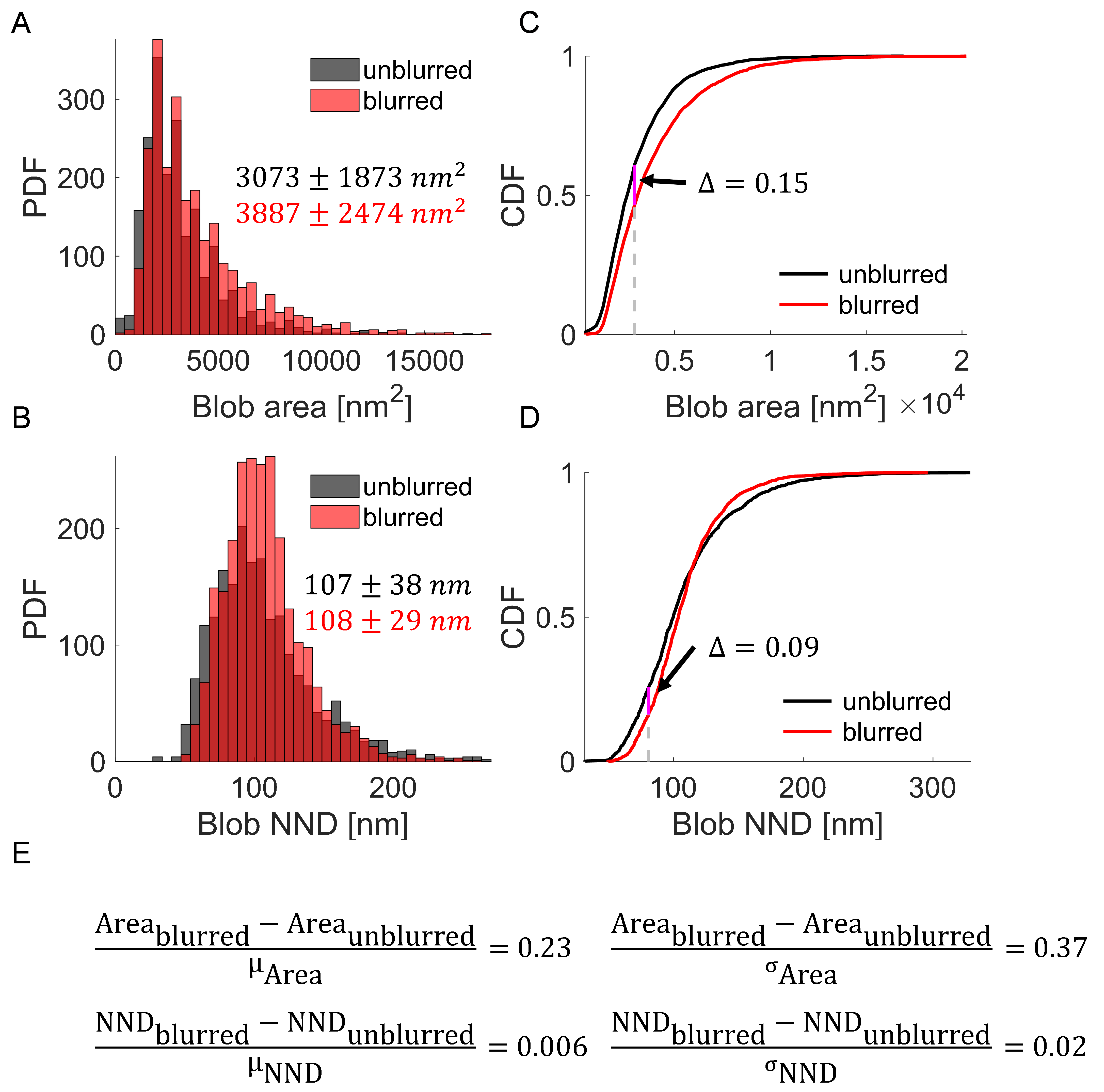

Supplement: Supplemental Material [file KNCL_A_1763093_SM8360.zip › Supplementary information/Figure_S3.png]
